# A preliminary anatomical study on carotid body of Makouei sheep 

**Published:** 2013

**Authors:** Gholamreza Najafi, Farhad Soltanalinejad, Hamed Hasanzadeh

**Affiliations:** 1 *Department of Anatomy and Embryology, Faculty of Veterinary Medicine, Urmia University, Urmia, Iran; *; 2 *Student of Veterinary Medicine, Urmia University, Urmia, Iran.*

**Keywords:** Anatomy, Carotid body, Makouei sheep

## Abstract

The carotid is a small mass of chemoreceptor's and sustentacular cells that detects changes in the composition of the arterial blood. The aim of the present study was to identify the size, color, location, blood and nerve supply of the carotid body in Makouei sheep. Fourteen heads of sheep from both sexes were collected from Urmia public slaughter-house. The exact situation and nerve supply of the carotid body was determined. Before dissection, blue latex was injected into right and left common carotid arteries. All the branches of the common carotid artery and the branch supplying carotid body were investigated. This study showed that, carotid body in sheep has been situated around the muscular branch of the occipital artery. The mean weight, width and length, thickness of carotid body were 0.01 g, 0.83 mm, 1.07 mm, and 1.06 mm respectively. Blood to the carotid body was supplied by glomic artery which was a branch of occipital artery. It was innervated by herring nerve which was a branch of glossopharyngeal nerve.

## Introduction

In 1743, carotid body was first described by Won Haller. It is a round, reddish-brown to tan structure found in adventitia of common carotid artery. In human, it is located on the posteromedial wall of the vessel at its bifurcation and is attached by Mayer's ligament through which the feeding vessels run.^1^ The carotid body is derived from the third branchial arch.^2^ In carotid body, type 1 cells act as peri-pheral chemoreceptors which detect changes in arterial partial pressure of O_2_ and Co_2_, pH fluctuations and helps maintain homeostasis via reflexive control of ventilation.^3^ Factors like temperature, osmolarity and arterial pressure, at least in animals, can stimulate the carotid body.^4^ There are two types of carotid body, compact and disseminated, compact part as in human,^1^ cat,^5^ hedgehog,^6^ and camel.^7^ However, few animals have both two types of carotid body, such as sheep and goat.^8^ The carotid body of goat was found to be a small oval or rounded parenchymatous organ. The carotid body was characterized by its profound vascularity.^9^

The carotid body of cattle,^10^ goat and sheep^8^ was found around muscular branch of the occipital artery. It was oval in shape and pink in color. Blood and nerve supply of carotid body was by occipital artery called the glomic artery, herring nerve which is a branch of glossopharyngeal nerve, respectively. The carotid body of the horse is small nodule present in the angle of common carotid artery division.^11^ It is enclosed in a fibrous sheath, and accompanied dorsally by the vagus and sympathetic nerves and ventrally by the recurrent laryngeal nerve. In the caudal part of the neck, it is in contact superficially with the external jugular vein, but further cranially the omohyoideus intervenes between the common carotid artery and the preceding vein. In birds, carotid body was located between the distal ganglion of the vagus nerve and recurrent laryngeal nerve at the beginning of the common carotid artery. In camels, it was located the point of separation of the internal carotid artery from the common carotid trunk.^7^

The aim of this study was to identify the exact location, blood and nerve supply of carotid body in Makouei sheep.

## Materials and Methods

In this study, 14 head of adult (1.5 to 2 years old) Makouei sheep from both sexes (7 male and 7 female) were used. The heads were collected from Urmia public slaughter-house. Before dissection, blue latex was injected into right and left common carotid artery. By dissection, location of bifurcation of common carotid artery were revealed. Then adventitial fat and connective tissue of external carotid and occipital arteries and muscular branch of occipital artery were removed from those vessels for clarification of carotid body. By dissection, nerve and blood supply of the carotid body was determined. The carotid body was weighted by digital balance. Maximum length, width and thickness of left and right side carotid body were measured by digital caliper under a stereomicroscope (Model SZX-ILLB200, Olympus Co., Tokyo, Japan). Data analysed with Student's *t-*test in SPSS (Version 16, IBM Company, Chicago, IL, USA). 

## Results

**Fig. 1:**
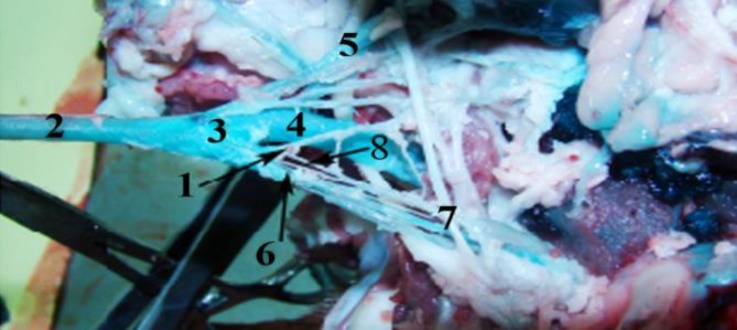
Lateral view of head after dissection in male Makouei sheep.

**Fig. 2:**
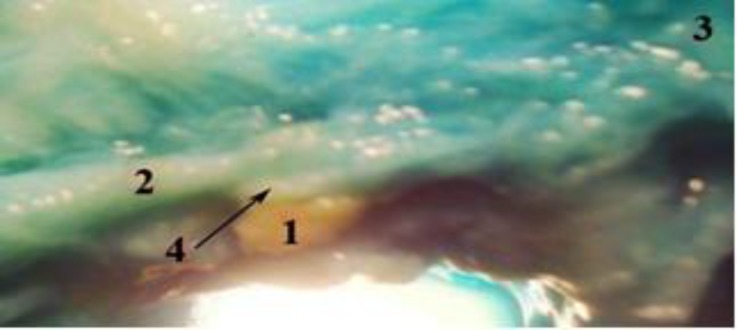
Photomicrograph of lateral view of head after dissection in male Makouei sheep (prepared by stereomicroscope, 25×).

In Makouei sheep (female and male), the carotid body (Glomus caroticum) on both sides were compact and ovoid situated at the bifurcation of the common carotid artery at around the muscular branch of the occipital artery. The carotid body was accompanied dorsally by glossopharyngeal nerve (the ninth cranial nerve) and ventrally by the occipital artery. In some cases, main mass of carotid body had several (2-3) distinct lobes at the around of muscular branch of the occipital artery. The carotid body was yellowish-brown in color and surrounded with a white loose connective tissue capsule. The present study revealed that carotid body was attached by dense connective tissue to muscular branch of occipital artery (Figs. 1 and 2). In this study, internal carotid artery was permutated in adult sheep. The blood the carotid body was supplied by a small branch of the occipital artery called glomic artery. The carotid body was innervated by herring nerve which is branch of glossopharyngeal nerve (Fig. 1). The mean weight, width and length, thickness of carotid body in the male and female sheep were shown in Table 1. There were no significant differences between all parameters in male and female sheep, (*p* > 0.05). 

1. Carotid body

2. Common carotid artery

3. Carotid sinus

4. External carotid artery

5. Lingual artery

6. Occipital artery

7. Glossopharyngeal nerve

8. Herring nerve

1. Carotid body

2. Muscular branch of occipital artery

3. Occipital artery

4. Dense connective tissue

**Table 1:** Weight, length, width and thickness of the carotid body in adult Macoee sheep (Mean ± SE).

**Factors**	**Male **		**Female**	
	**Right**	**Left**	**Right**	**Left**
**Weight** ** (g)**	0.01 ± 0.00	0.01 ± 0.00	0.01 ± 0.00	0.01 ± 0.00
**Width** ** (mm)**	1.01 ± 0.01	0.97 ± 0.02	0.76 ± 0.01	0.57 ± 0.01
**Length** ** (mm)**	1.03 ± 0.03	1.22 ± 0.03	1.06 ± 0.03	0.95 ± 0.01
**Thickness (mm)**	0.84 ± 0.01	1.00 ± 0.02	1.22 ± 0.03	1.19 ± 0.03

No significant differences were found between male and female (*p* > 0.05).

## Discussion

The morphological structure of the carotid body of the sheep is similar to that of other ruminants (camel, goat, and cattle).^7^^,^^8^^,^^10^ The carotid body in adult sheep was found close to the origin of the muscular branch of the occipital artery. Carotid body was ovoid and yellowish-brown in color. These findings agree with the results as regards cattle.^10^ These findings differ from results of studies in the other animals that having internal carotid artery i.e., camel,^7^ horse,^11^ rabbits,^12^ and birds.^13^ Internal carotid artery per-muted in sheep similar to cattle.^10^ Therefore, in present study carotid body located at the origin of muscular branch of the occipital artery. In this study blood to the carotid body was supplied by the fine branch of the occipital artery (glomic artery) similar to that of cattle. The carotid body in sheep is innervated by herring nerve which is a short branch of the glosso-pharyngeal nerve. These findings conformed to that of other mammalian species.^8^^,^^10^
